# MMP3 activity rather than cortical stiffness determines NHE1-dependent invasiveness of melanoma cells

**DOI:** 10.1186/s12935-019-1015-7

**Published:** 2019-11-09

**Authors:** Dennis Keurhorst, Ivan Liashkovich, Fabian Frontzek, Svenja Nitzlaff, Verena Hofschröer, Rita Dreier, Christian Stock

**Affiliations:** 1 Institute of Physiology II, University of Münster, Robert-Koch-Str. 27b, 48149 Münster, Germany; 2 Department of Oncology and Hematology, University Hospital of Münster, Albert-Schweitzer-Campus 1, 48149 Münster, Germany; 3 Institute of Animal Physiology, University of Münster, Schlossplatz 8, 48143 Münster, Germany; 4 Institute of Physiological Chemistry and Pathobiochemistry, University of Münster, Waldeyer-Str. 15, 48149 Münster, Germany; 5 Department of Gastroenterology, Hannover Medical School, Carl-Neuberg-Str. 1, 30625 Hannover, Germany

**Keywords:** Cell migration, (Cortical) cell stiffness, Matrix digestion, Metastasis, pH

## Abstract

**Background:**

Both cell adhesion and matrix metalloproteinase (MMP) activity depend on pH at the cell surface. By regulating extracellular juxtamembrane pH, the Na^+^/H^+^ exchanger NHE1 plays a significant part in human melanoma (MV3) cell migration and invasion. Because NHE1, besides its pH-regulatory transport function, also serves as a structural element tying the cortical actin cytoskeleton to the plasma membrane, we investigated whether NHE1 affects cortical stiffness of MV3 cells, and how this makes an impact on their invasiveness.

**Methods:**

NHE1 overexpressing MV3 cells were compared to the corresponding mock-transfected control cells. NHE1 expression was verified by Western blotting, cariporide (HOE642) was used to inhibit NHE1 activity, cell stiffness was determined by atomic force microscopy, and F-actin was visualized by phalloidin-staining. Migration on, and invasion of, native and glutaraldehyde-fixed collagen I substrates were analyzed using time-lapse video microscopy and Boyden-chamber assays, respectively. MMP secretion and activity were detected by Western blot and zymography, respectively. MMP activity was inhibited with NNGH.

**Results:**

The cortical, but not the bulk stiffness, was significantly higher in NHE1 overexpressing cells. This increase in cortical stiffness was accompanied by a reorganization of the cortical cytoskeleton, i.e. a condensation of F-actin underneath and along the plasma membrane. However, it was not affected by NHE1 inhibition. Nevertheless, actin dynamics is required for cell invasion as demonstrated with the application of cytochalasin D. NHE1 overexpression was associated with an elevated MMP3 secretion and an increase in the invasion of a native matrix. This increase in invasiveness could be antagonized by the MMP inhibitor NNGH. Transmigration through a glutaraldehyde-fixed, indigestible substrate was not affected by NHE1 overexpression.

**Conclusion:**

NHE1, as a structural element and independently of its transport activity, contributes to the organization of the cortical F-actin meshwork and thus impacts cortical stiffness. Since NHE1 overexpression stimulates MMP3 secretion but does not change transmigration through a fixed substrate, MV3 cell invasion of a native substrate depends on MMP activity rather than on a modifiable cortical stiffness.

## Background

Upregulation of the expression/activity of the ubiquitously expressed acid-extruding membrane transporter NHE1 (Na^+^/H^+^ exchanger 1) has been commonly correlated with tumor malignancy [1]. NHE1, by taking advantage of the inwardly directed Na^+^-gradient across the plasma membrane, exchanges one H^+^ for one Na^+^ and thus contributes significantly to cellular pH homeostasis [2]. A higher basal NHE1 activity is characteristic of tumor cells. It often leads to an increase in cytosolic steady state pH_i_, and an alkaline pH_i_ favors (aerobic) glycolytic metabolism [3, 4], proliferation and evasion of apoptosis [5]. The resulting decrease in extracellular pH (pH_e_), especially when accompanied by local intracellular alkalization [6], promotes tumor cell migration and invasion [7]. The intracellular pH affects cytoskeletal dynamics while the pH at the cell surface modulates cell/matrix interactions and stimulates the activity of matrix metalloproteinases (MMPs) [8]. Thus, by regulating pH_i_ and pH_e_, NHE1 activity has a significant effect on the three major variables underlying cell motility: (i) MMPs clearing the way through the extracellular matrix, (ii) focal adhesion complexes ensuring a well-balanced substrate grip, and (iii) the cytoskeletal machinery including actomyosin dynamics considered as the engine for cell migration.

In addition to its pH- and osmoregulatory transport function, NHE1 (i) operates as a plasma membrane scaffold in the assembly of signaling complexes and (ii) serves as a structural anchor for actin filaments through its direct binding of actin binding proteins of the ezrin, radixin and moesin (ERM) family [9]. As an actin anchoring protein, NHE1 maintains the cell shape by tying the plasma membrane to cortical actin filaments [10]. H^+^ export and actin anchoring, both mediated by NHE1, dynamically coordinate the remodeling of actin and cell-substrate adhesion, which considerably contributes to cell motility [11].

The concerted action of actin and its accessory and regulatory proteins such as non-muscle myosin II is required not only for cell migration and invasion, but also for defining and modulating the cell shape [12]. Accordingly, the cortical actin-myosin network, located right underneath the plasma membrane, determines the mechanical properties of the cell surface. Structure, density and integrity of the cortical actin meshwork determine cortical elasticity or stiffness [13] and can be affected by various parameters including the plasma membrane potential [14].

Local rupture or regulated tapering of the cortical actin network can lead to the formation of blebs. Usually, extensive blebbing indicates apoptosis, but the formation of blebs can also be characteristic of an amoeboid mode of cell migration, particularly of an invasive behavior in a spatially constrained environment [15, 16]. In this setting, blebs seem to be advantageous and replace lamellipodia and other protruding structures [17]. Consistently with the occurrence of blebbing due to local changes in the cortical actin network during amoeboid movement in a dense extracellular matrix network, the stiffness of both tumor cells obtained from patients and cancer cell lines has been shown to inversely correlate with the invasion of three-dimensional basement membranes. Cancer cells with the lowest invasive potential are five times stiffer than those with the highest invasive potential [18]. This holds true also for ovarian cancer cells that are generally softer than non-malignant ovarian epithelial cells [19] and for cancerous human bladder cells [20].

As NHE1 is able to tie the cortical actin to the membrane, and the integrity of the cortical actin network affects stiffness, blebbing and invasiveness, the present study aims to investigate a possible relationship between NHE1 expression, cell stiffness and invasiveness.

## Material and methods

### Cell culture

Human melanoma cells of the MV3 cell line [21], stably transfected with an empty pcDNA3 vector (control) or with the pcDNA3 vector carrying NHE1 (NHE1 overexpressing; [22]), were grown in bicarbonate buffered Roswell Park Memorial Institute (RPMI) 1640 medium (Sigma, Taufkirchen, Germany) supplemented with 10% (*v/v*) fetal calf serum (FCS) at 37 °C in a humidified atmosphere of 5% CO_2_, 95% air. The culture medium contained 0.6 g l^−1^ geneticin (G-418-sulfate; PAA Laboratories, Pasching, Austria) in order to select the transfected cells.

### Detection of NHE1 and MMP3 by Western blot

#### NHE1

Confluent cell cultures were washed with cold Dulbecco’s phosphate-buffered saline (PBS w/o Ca^2+^, Mg^2+^; Sigma-Aldrich) and lysed at 4 °C in radioimmunoprecipitation assay (RIPA) lysis buffer (150 mmol l^−1^ NaCl, 25 mmol l^−1^ Tris HCl (pH 7.6), 1% Nonidet P-40, 0.1% SDS, 1.0% sodium deoxycholate, a protease and a phosphatase inhibitor cocktail (cOmplete, Mini; PhosSTOP; both from Roche)). Lysates were scraped off and spun down at 13,000×*g* and 4 °C for 10 min. Protein concentrations were determined with the Bicinchoninic Acid Protein Assay Kit (Thermo Scientific). Equal amounts of protein (~ 30 µg) mixed with sample buffer (4:1 (*v/v*); 500 mmol l^−1^ Tris, 100 mmol l^−1^ dithiothreitol, 8.5% SDS, 27.5% sucrose, and 0.03% bromphenol blue indicator) were loaded, separated by SDS-PAGE (7.5% acrylamide gels; Minigel System, Bio-Rad Laboratories) and transferred onto polyvinylidene difluoride (PVDF) membranes (Immobilon Transfer Membranes, Millipore) by tank blotting at 4 °C overnight. PVDF membranes carrying the blotted proteins were immersed in 5% (w/v) skim milk in 0.05% (v/v) Tween in PBS Dulbecco (w/o Mg^2+^; Biochrom AG) for 30 min at room temperature followed by overnight incubation with the primary antibody against NHE1 (mouse, 1:1000 in 5% skim milk/0.05% PBS-T; BD Biosciences). After washing (3 × 10 min in 0.05% Tween in PBS), blots were incubated for 1 h with a peroxidase (POD)-conjugated secondary antibody (goat anti-mouse POD, 1:25,000 in 5% skim milk/0.05% PBS-T, Dianova) and then washed again (3 × 10 min in 0.05% Tween in PBS). Blots were developed using a chemiluminescence kit (SuperSignal West Femto Maximum Sensitivity Substrate, Thermo Scientific). Autoluminography was carried out with a ChemiDoc XRS gel documentation system and Quantity-One analysis software (Bio-Rad Laboratories). To control protein loading, membranes were stripped and then probed with a monoclonal anti-β-actin antibody (anti-mouse, 1:10,000; Sigma Life Science; secondary antibody 1:25,000 (goat anti-mouse POD, Dianova)). The Quantity-One software (Bio-Rad) was applied for densitometric analyses. The protein amount was normalized to the amount of β-actin.

#### MMP3

Confluent cell cultures were thoroughly washed with PBS (Dulbecco, Biochrom AG) and then kept in serum-free, G418-containing RPMI 1640 medium for 24 h. In order to inhibit NHE1 activity, cariporide (HOE642; Santa Cruz Biotechnology; final concentration 10 µmol l^−1^) was added and renewed after 12 h. After 24 h, 1 ml of the MMP-containing medium was used for trichloroacetic acid (TCA) precipitation. After adding 225 µl 60% TCA (final concentration ~ 12%) and 139 µl 1%Triton (final concentration ~ 0.1%), the medium was vortexed and incubated on ice for 20 min. The precipitate was spun down at 14,000 rpm, 4 °C for 20 min, and washed twice with EtOH for 30 min. Each washing step was followed by 20 min centrifugation. Finally, the pellet was rinsed with ice cold acetone, centrifuged for 20 min, dried at 37 °C, resuspended and vortexed in sample buffer (2% SDS, 10% Glycerol, 60 mmol l^−1^ Tris–HCl (pH6.8), 0.001% bromophenol blue, 5% β-mercaptoethanol), and then heated to 95 °C for 5 min. 40 µl samples were subjected to electrophoresis in 4.5–15% SDS–polyacrylamide gradient-gels run at 30 mA. The separated proteins were electrotransferred to a nitrocellulose membrane (Protran nitrocellulose transfer membrane, BA 83, 0.2 µm, Whatman plc) at a constant current of 80 mA for 3 h at 4 °C. Total protein detection served as a loading control and was performed using the Pierce MemCode™ reversible protein stain kit (Pierce Biotechnology Inc.). The nitrocellulose membrane was destained using the MemCode stain eraser. Unspecific binding sites were blocked with Tris-buffered saline (TBS-T: 50 mmol l^−1^ Tris/HCl (pH7,4), 150 mmol l^−1^ NaCl, 0.05% Tween-20) containing 5% skim milk and 1% bovine serum albumin for 1 h at room temperature. The nitrocellulose membrane was then incubated with a primary antibody against MMP3 (monoclonal from rabbit (Abcam), 1:1000 in TBS-T containing 2.5% skim milk and 0.5% BSA) at 4 °C overnight. The nitrocellulose membrane was rinsed with TBS-T (3 × 15 min) followed by 1 h incubation with the secondary, horseradish peroxidase-conjugated antibody (donkey anti-rabbit (Amersham BioSciences), 1:10,000 in TBS-T with 2.5% skim milk and 0.5% BSA) at room temperature. After washing with TBS-T (3×), the membrane was developed employing the SuperSignal West Femto Maximum Substrate (Thermo Fisher Scientific). Detection of labeled protein bands was performed with a chemiluminescence imaging system (Fusion SL4.2P, Vilber Lourmat).

### Actin staining

Cells were seeded onto collagen type I-coated (Collagen G, Biochrom AG; final concentration 0.4 mg ml^−1^) coverslips and cultured for 2 h. The cells were then fixed with 3.5% paraformaldehyde (*w/v*) in PBS (Dulbecco, Biochrom AG) for 30 min and permeabilized for 25 min in 0.1% (*v/v*) Triton X-100/TBS in order to ensure that the intracellular F-actin epitopes were accessible to the antibody. After washing with PBS (2×), nonspecific binding sites were blocked with 3% bovine serum albumin (BSA) in PBS (*w/v*) for 2 h at room temperature. The cells were then stained with Alexa Fluor® 488 Phalloidin (Invitrogen AG; dilution 1:100) for 45 min. Prior to Dako mounting (Dako A/S, Glostrup, Denmark), the cells were washed in PBS once again. Images were taken with a digital camera (Model 9.0, RT-SE-Spot, Visitron Systems, Puchheim, Germany) fitted to an inverted microscope (Axiovert 200, Carl Zeiss AG) and controlled by MetaVue software (Visitron Systems).

### Measuring cell stiffness by atomic force microscopy (AFM)

Employing a JPK NanoWizard 3 (JPK Instruments, Berlin, Germany) combined with a Leica DMI 6000 (CS Trino AFC) inverted fluorescence microscope (Leica Microsystems GmbH, Wetzlar, Germany) and the JPK SPM software (JPK Instruments) both the cortical and the bulk stiffness of the two MV3 clones were measured. To this end MV3 cells were seeded onto collagen type I (Collagen G, Biochrom AG)-coated glass bottom dishes (35 mm in diameter, WillCo Wells) at a density corresponding to ~ 70% confluency and were kept in HEPES buffer (122.5 mmol l^−1^ NaCl, 5.4 mmol l^−1^ KCl, 0.8 mmol l^−1^ MgCl_2_, 1.2 mmol l^−1^ CaCl_2_, 5.5 mmol l^−1^ glucose, 10.0 mmol l^−1^ HEPES; pH 7.4) at 37 °C in a heated chamber mounted on the stage of the microscope. For the AFM-measurements a soft cantilever (nominal spring constant = 0.03 N m^−1^, Novascan Technologies) with a spherical tip (sphere diameter = 10 µm) and a maximum loading force of approximately 1 nN were used.

Essential mechanical probing parameters including deflection sensitivity and cantilever spring constant were calibrated prior to each experiment. Cell stiffness was chosen as a preferred quantitative readout of mechanical alteration of the cells. The stiffness value provides the most direct and straightforward mechanical readout for a given combination of an AFM probe and a sample. On the other hand, such precise calculation of the elastic modulus relies heavily on several additional parameters [23] which could not be obtained during our experiments. Cell nanoindentation was performed by applying a maximal loading force of 1 nN at a loading rate of 1 µm s^−1^. Resulting cell deformation was used to calculate the cell stiffness as described previously [24].

### Preparation of collagen matrices for cell migration

A collagen I substrate was prepared by gently mixing 210 µl of 5× RPMI1640, 210 µl of 5× HEPES-buffer (final concentration in the polymerized collagen gels: 10 mmol l^−1^), 245 µl of distilled water and 430 µl of Collagen G (containing acid-soluble calfskin collagen type I at a concentration of ~ 4 mg ml^−1^). The pH value of the mixture was adjusted to 7.4 with 1 M NaOH. A thin layer (~ 200 µl) of this collagen I matrix polymerized on the bottom of a 12.5 cm^2^ culture flask (Falcon, Corning Inc.) in a humidified atmosphere at 37 °C overnight. The next day, the cells were seeded onto this matrix and allowed to attach and spread for 6 h.

In one set of experiments matrix metalloprotease (MMP) activity was disabled by fixing the matrix with 2 ml of 2% glutaraldehyde in PBS (*v/v*) for 15 min. The matrix was washed (5 × 5 min) with PBS and stored in PBS at 4 °C overnight. The next day, the fixed matrix was washed again (5 × 5 min PBS) and the cells were seeded.

### Cell migration

The culture flasks were placed in heated chambers (37°) on stages of inverted microscopes (Axioverts 40 C and 20, Carl Zeiss AG). Employing video cameras (Model XC-ST70CE and XC-77CE; Hamamatsu/Sony) and PC-vision frame grabber boards (Hamamatsu) cell migration was recorded in 10 min intervals for 5 h. Images were acquired with HiPic and WASABI software (Hamamatsu), and cell contours were labeled applying AMIRA software (TGS, Template Graphics Software, Mercury Communication System Inc.). From these contours the migration velocity (µm min^−1^), translocation (µm), total distance covered (µm), cell area (µm^2^) and the structural index (SI) were analyzed using the NIH ImageJ software and self-made Java programs [25]. Migratory speed was determined from the movement of the cell center, translocation corresponds to the linear or net distance covered, and SI represents the morphological cell shape. SI was calculated according to the formula SI = (4π*A*)/*p*^*2*^, where *p* represents the perimeter of the area *A* covered by the cell. A spherical cell is represented by values close to 1, a dendritic cell shape by values close to 0. A directionality index (di) was calculated as:

$$di = \frac{{linear\;\; distance \;\;covered \left( {\upmu {\text{m}}} \right)}}{{mean \;velocity \left( {\upmu {\text{m}}/{ \text{min} }} \right) \times total\;\;duration (\text{min} )}}.$$

### Invasion–transmigration

Transmigration was determined employing Boyden chamber assays. 20 µl of the collagen I mixture (composition as described above) were allowed to polymerize on a filter-membrane (insert for a 24 well plate, 8.0 µm pore size; ThinCert, Greiner Bio-One GmbH) at 37 °C in a humidified atmosphere overnight. 200,000 cells per filter were seeded onto this collagen matrix. After 24 h incubation in RPMI1640 with G-418 and serum, the medium was gently renewed for another 24 h. Cells were then fixed and stained with crystal violet (Sigma-Aldrich) in PBS. The matrix and the remaining cells on the upper side of the filter were removed and excess crystal violet was washed away with PBS. The invasive cells that remained on the lower side of the filter and those on the bottom of the well were counted.

MMP activity was inhibited by 10 µmol l^−1^ NNGH (*N*-isobutyl-*N*-(4-methoxyphenylsulfonyl)glycyl hydroxamic acid; Sigma-Aldrich), and NHE1 was inhibited with 10 µmol l^−1^ cariporide (HOE642). DMSO, the solvent for NNGH and cariporide, reached a final concentration of 0.1%.

Total MMP activity was disabled by fixing the matrix with 1 ml of 2% glutaraldehyde in PBS (*v/v*) for 15 min. In order to ascertain the role of actin in transmigration through a fixed matrix, cells were exposed to Cytochalasin D (50 nmol l^−1^) over the entire experiment (48 h).

### *Matrix digestion* in situ

20 µl of the collagen mixture (see above) were allowed to polymerize on coverslips (ø 15 mm, R. Langenbrinck GmbH, Germany) for at least 3 h in a humidified atmosphere (5% CO_2_, 95% air) at 37 °C. The matrices were then either kept in PBS at 4 °C until use, or they were fixed with 2% glutaraldehyde in PBS (*v/v*) for 15 min, washed (5 × 5 min) with PBS and stored in PBS at 4 °C overnight. The next day, the fixed collagen substrates were washed again (5 × 5 min PBS), and cells were seeded onto the fixed and the native matrices. The medium was renewed after 24 h. After 48 h the cells on the native matrix were fixed with 2% glutaraldehyde in PBS (*v/v*) for 15 min and then washed with PBS (5 × 5 min). The coverslips carrying the cells were mounted on glass slides with fluorescence mounting medium containing DAPI (4′,6-diamidino-2-phenylindol; Dako A/S, Glostrup, Denmark). The glutaraldehyde-induced autofluorescence of the collagen substrate (at 488 nm) was evaluated employing the same setup as that used for the actin staining. The NIH ImageJ software (http://rsb.info.nih.gov/ij/) was used to determine the fluorescence intensity per visual field (233.086 pixels).

### Zymography

20 µl of conditioned media from confluent cell cultures were mixed with equal volumes (20 µl) of twofold concentrated sample loading buffer (2 mmol l^−1^ EDTA, 2% SDS, 20% glycerol, 0.02% bromophenol blue, 20 mmol l^−1^ Tris/HCl, pH 8.0) and subjected to electrophoresis on a 1% gelatin-(porcine skin, Sigma-Aldrich) containing 4.5–15% gradient SDS–polyacrylamide gel run at 40 mA. The gel was washed twice for 30 min in 2.5% Triton-X 100, rinsed in distilled water and then developed in 50 mmol l^−1^ Tris/HCl (pH 8.5) containing 5 mmol l^−1^ CaCl_2_ overnight at 37%. It was stained with Coomassie brilliant blue R250 (0.15% Coomassie BB R-250; Bio-Rad Laboratories).

### Statistics

Data are presented as the mean values ± SEM. Depending on the experiment type experiments were repeated three up to nine times. Significance of the data was determined with the student’s unpaired or paired t test. *p* < 0.05 was set as the level of significance (*p < 0.05; **p < 0.01; ***p < 0.001).

## Results

### Expression of NHE1

**Fig. 1:**
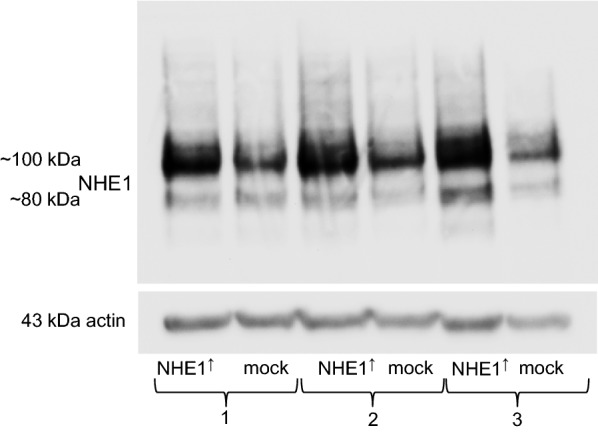
NHE1 expression. Expression of the glycosylated NHE1 molecule (~ 110 kDa) and the NHE1 precursor (~ 85 kDa) in NHE1 overexpressing MV3 cells and the corresponding mock controls. Three pairs of lysates (1–3) from three independent cell culture preparations (*N *= 3) are shown

Differences in the expression of NHE1 in control (empty vector) and NHE1 overexpressing MV3 cells, previously generated by Frontzek et al. (2014), were checked by Western blot (Fig. 1). The expression of both, the glycosylated NHE1 molecule (~ 110 kDa) and the NHE1 precursor (~ 85 kDa), was clearly increased in NHE1 overexpressing cells.

### Cortical stiffness depends on NHE1 expression

**Fig. 2:**
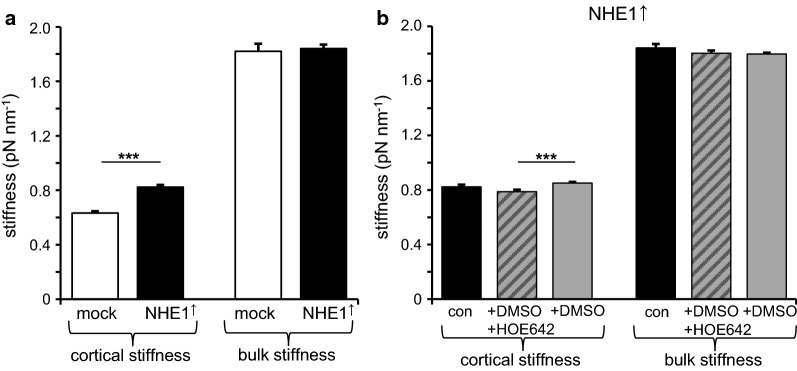
Cortical stiffness depends on NHE1 expression. **a** The cortical stiffness of NHE1 overexpressing MV3 cells (n = 123 cells from *N *= 4 independent experiments) is significantly higher than that of the mock controls (n = 141, *N *= 3) while the bulk stiffness is not affected (n = 119, *N *= 4 for NHE1 overexpressing cells; n = 143, *N *= 3 for empty vector controls). **b** The bulk stiffness of NHE1 overexpressing MV3 cells is not affected by NHE1 inhibition with cariporide (HOE642). Although there is no difference in cortical stiffness between entirely untreated (n = 119, *N *= 4) and cariporide treated (n = 93, *N *= 3) cells, a slight, yet significant difference can be observed between cells treated with the solvent DMSO alone (n = 144, *N *= 3) and those treated with cariporide dissolved in DMSO

NHE1 is bound to the cortical actin cytoskeleton by adaptor proteins such as members of the ERM family [26]. Hence, the question arises as to whether or not the cortical cell stiffness might be affected by the presence and/or activity of NHE1. By employing AFM the cortical and the bulk stiffness of MV3 cells were determined. The cortical stiffness was significantly higher in NHE1 overexpressing than in control cells whereas the bulk stiffness did not differ (Fig. 2a). Neither the cortical nor the bulk stiffness were substantially decreased when NHE1 activity was inhibited by cariporide (HOE642) (Fig. 2b) indicating that the mere presence of NHE1 as a structural element has an impact, independently of its ion transport function. However, there was a significant difference in cortical stiffness between cells treated with cariporide and those exposed to DMSO as the solvent for cariporide, suggesting that DMSO alone may have an effect on the cortical stiffness.

### NHE1 expression affects F-actin

**Fig. 3:**
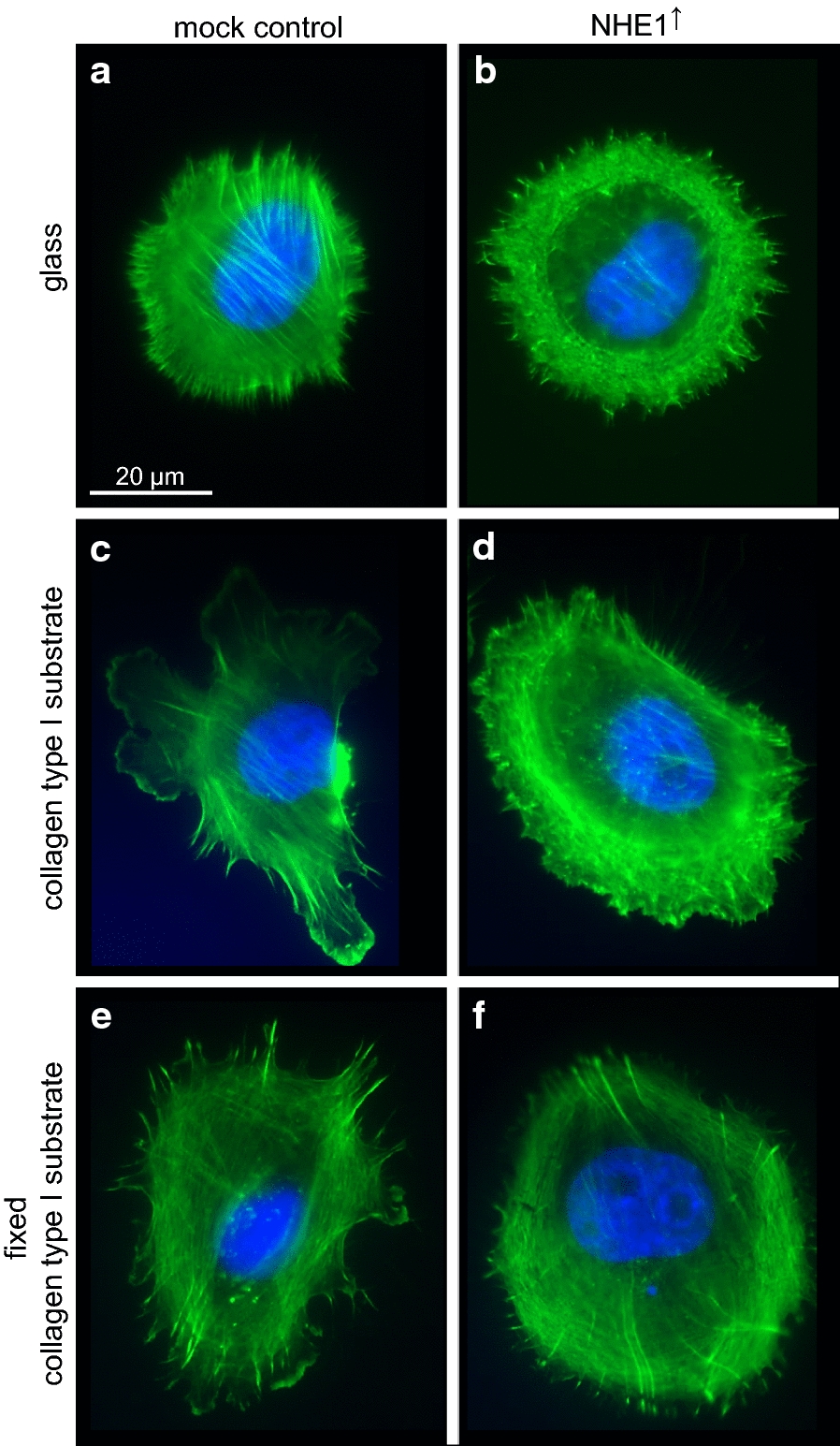
F-actin arrangement depends on NHE1 expression. Regardless of whether MV3 cells were seeded on glass (**a**, **b**), collagen type I (**c**, **d**) or a glutaraldehyde-fixed collagen type I substrate (**e**, **f**), NHE1 overexpression (**b**, **d**, **f**) is accompanied by a rearrangement of F-actin. In addition, the cells surface is characterized by the occurrence of numerous, actin-containing spike-like structures. Actin was labelled with phalloidin. Scale bar: 20 µm

The finding that NHE1 overexpressing cells show a higher cortical stiffness prompted us to visualize cortical F-actin by phalloidin staining. Indeed, NHE1 overexpression was associated with a rearrangement of cortical F-actin (Fig. 3). Whether seeded on glass, a native or a glutaraldehyde-fixed collagen type I matrix, NHE1 overexpressing cells always showed a higher cortical actin density and less stress fibers than the empty vector controls. The F-actin appeared as a belt-like structure, possibly forming a thick layer of cortical F-actin at the expense of stress-fibers. In addition, NHE1 overexpression caused the formation of spike-like structures at the cell surface and an annular arrangement of the F-actin (Fig. 3b, d, f). This observation suggests a positive correlation between the NHE1 expression level, the annular arrangement of cortical F-actin and the strength of the cortical actin skeleton.

### NHE1 expression affects matrix digestion

**Fig. 4:**
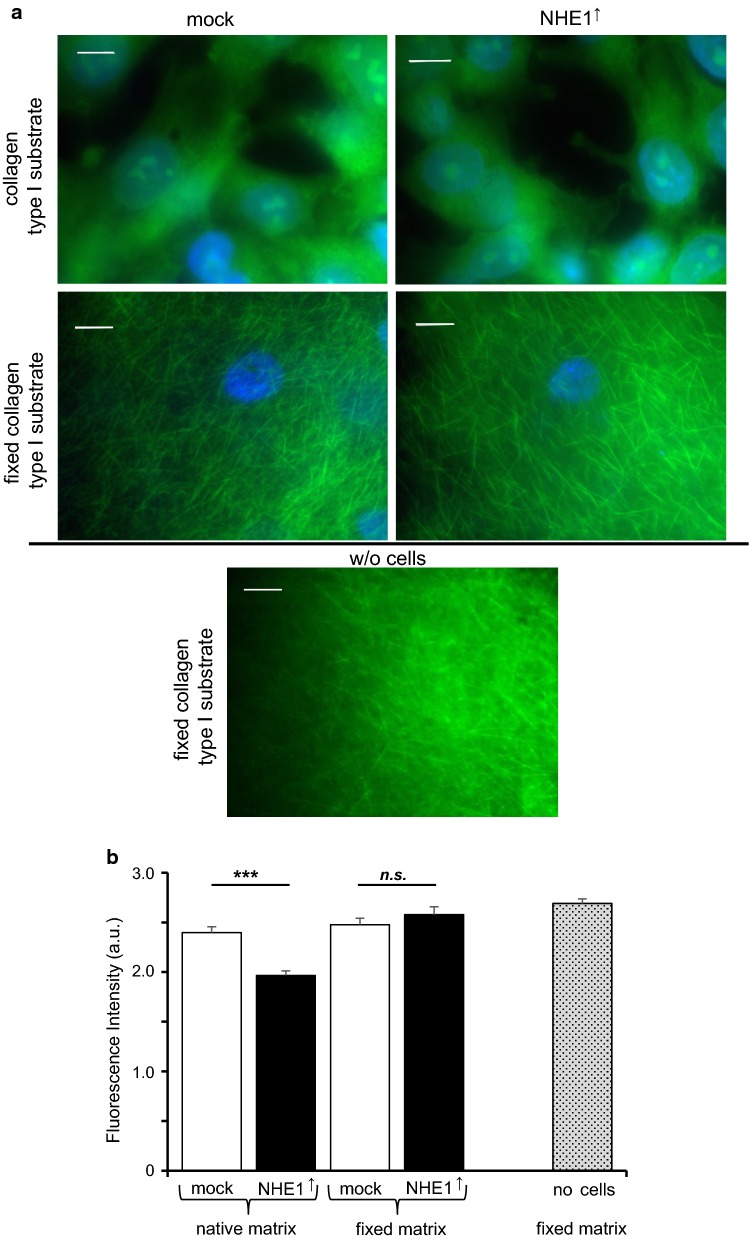
Digestion of collagen type I is fostered by NHE1 expression. **a** Fluorescence images of MV3 cells on native (upper images) and fixed collagen type I substrate (second row). The lowest image shows a fixed collagen type I substrate without (w/o) cells. Cells were kept on the respective substrate for 48 h and then fixed with glutaraldehyde. While the fixed substrate is characterized by a network of collagen I fibers the native substrate has been remodeled and mostly digested. Blue: DAPI staining of nuclei; green: glutaraldehyde-induced autofluorescence. Scale bar: 10 µm. **b** Fluorescence intensity measurements of native and fixed matrices. Native matrices populated with NHE1 overexpressing MV3 cells (n = 49 areas from *N *= 4 independent experiments) show a significantly lower intensity than those with control cells (n = 25, *N *= 2). Regardless of whether being populated with NHE1 overexpressing (n = 40, *N *= 9) or control cells (n = 15, *N *= 3), the intensities of the fixed substrates did not differ from that of unsettled matrices (n = 18, *N *= 3)

Since the activity of NHE1 has been shown to favor MMP-mediated ECM-digestion and thus invasion [27], mock-control and NHE1 overexpressing cells were compared regarding their digestive activity on a native and a glutaraldehyde-fixed reconstituted collagen matrix. The glutaraldehyde-induced autofluorescence was taken advantage of in order to identify low-matrix or matrix-free regions that represent MMP activity. While there was no noteworthy ECM-digestion on the fixed matrix, NHE1 overexpressing cells digested significantly more (~ 16%) of the native matrix than the mock-control cells (Fig. 4).

### NHE1 overexpression reduces cell migration on fixed collagen type I

**Fig. 5:**
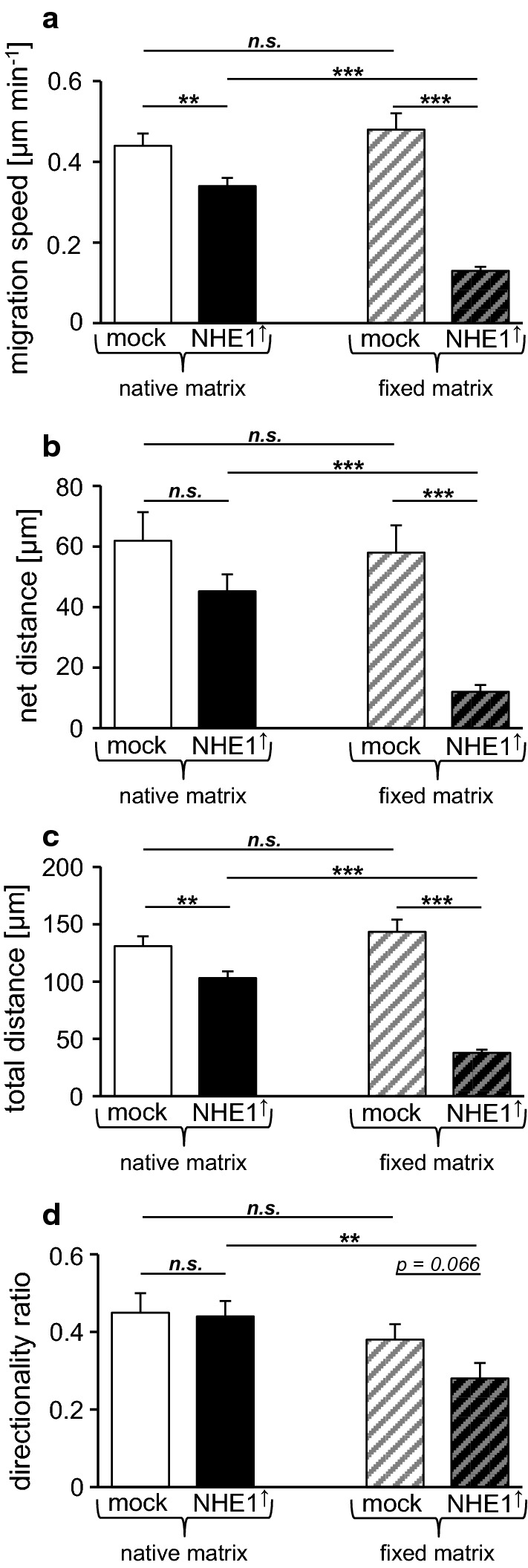
MV3 cell migration is affected by NHE1 expression and matrix fixation. The effects of NHE1 overexpression become more apparent on a fixed collagen type I substrate. **a** The migration speed of NHE1 overexpressing cells is significantly lower than that of control cells, on both the native and the fixed substrate. NHE1 overexpressing cells migrate significantly more slowly on the fixed (n = 41 cells from *N *= 3 independent experiments) than on the native substrate (n = 40 cells, *N *= 3) while control cells do not migrate differently (n = 33, *N *= 3 on fixed vs. n = 30, *N *= 5 on native). **b** The net distance covered within 5 h is lower in NHE1 overexpressing cells with a significant difference on the fixed substrate. NHE1 overexpressing cells cover a shorter distance on the fixed than on the native substrate while the distance covered by control cells does not differ between the two substrates. **c** NHE1 overexpressing cells cover a lower total distance on both substrates, when compared to the control cells. Compared to the native matrix, NHE1 overexpressing cells cover a lower total distance on the fixed matrix whereas control cells do not show a difference. **d** The directionality index does not differ between control and NHE1 overexpressing cells on the native substrate. On the fixed substrate, NHE1 overexpressing cells migrate less directional than control cells on the fixed and NHE1 overexpressing cells on the native substrate

Since the protons extruded by NHE1 are known to fine-tune the interplay between formation and release of focal adhesion contacts as required for cell migration [6, 28], we compared the migratory behavior of mock-control and NHE1 overexpressing cells on a fixed and a native collagen (type I) substrate (Fig. 5, Table 1). In control cells, substrate fixation did not cause changes in migratory speed (Table 1, *p *= 0.37), net distance (*p *= 0.75) total distance covered within 5 h (*p *= 0.37), and directionality (*p *= 0.24). In NHE1 overexpressing cells, however, matrix fixation led to significant decreases in all of the observed parameters [migratory speed (*p *< 10^−15^), net distance (*p *< 10^−6^), total distance (*p *< 10^−15^), and directionality (*p *= 0.004)]. Comparing control and NHE1 overexpressing cells reveals, that on both, the native and the fixed collagen substrate, NHE1 overexpressing cells migrate significantly more slowly (native matrix: *p *= 0.006; fixed matrix: *p *< 10^−15^) and generally cover shorter distances (net and total distance on native (net: *p *= 0.106; total: *p *= 0.006) and fixed matrix (net: p < 10^−6^; total: *p *< 10^−15^), respectively), while the directionality is hardly affected. The observed decrease in migratory activity could be caused by either an excessive or by an insufficient interaction between cell surface and substrate. In order to find out more about the possible reasons we analyzed the cells’ morphology.

**Table 1:** Effects of matrix fixation and NHE1 expression on cell migration and morphology

Matrix	Native		Fixed	
NHE1 expression	Mock control (n = 30; *N *= 5)	Overexpressed (n = 40; *N *= 3)	Mock control (n = 40; *N *= 3)	Overexpressed (n = 41; *N *= 3)
Speed [µm min^−1^]	0.44 ± 0.03	0.034 ± 0.02	0.48 ± 0.04	0.13 ± 0.01
Net distance [µm]	61.92 ± 9.42	45.17 ± 5.6	57.92 ± 9.07	11.93 ± 2.33
Total distance [µm]	130.92 ± 8.61	103.12 ± 5.78	143.37 ± 10.67	37.84 ± 2.83
Directionality	0.45 ± 0.05	0.44 ± 0.04	0.38 ± 0.04	0.28 ± 0.04
Structural index	0.43 ± 0.03	0.5 ± 0.02	0.3 ± 0.01	0.42 ± 0.02
Area [µm^2^]	888 ± 112	1115 ± 84	1251 ± 78	1298 ± 94

Data are shown as mean ± SEM For *p* values and further information, please see text

**Fig. 6:**
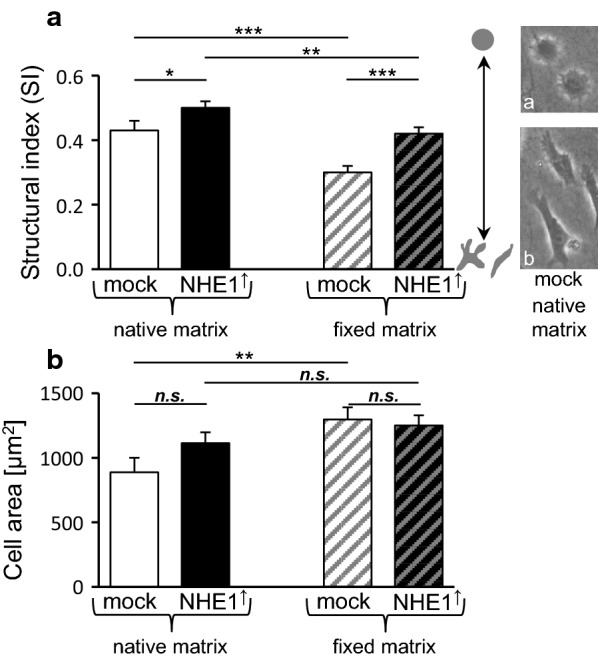
Morphological parameters of MV3 cells depend on NHE1 expression and matrix fixation. **a** While both NHE1 overexpressing and control cells are less spherical, i.e. more branched on the fixed substrate, NHE1 overexpressing cells are generally more spherical than the control cells. The images show control cells on native substrate, representing (**a**) spherical (SI values closer to 1) and (**b**) branched or spindle-shaped (SI values closer to 0) morphologies. **b** On both substrates, the cell area of control and NHE1 overexpressing cells is not different. However, control cells are significantly larger on the fixed than on the native substrate. NHE1 overexpressing cells on fixed (n = 41 from *N *= 3 independent experiments) and native substrate (n = 40, *N *= 3); mock control cells on fixed (n = 33, *N *= 3) and native substrate (n = 30; *N *= 5)

To a certain extent, the cell morphological parameters reflect the results obtained from the migration experiments (Fig. 6, Table 1). On both, the native and the fixed substrate, the NHE1 overexpressing cells were more spherical (Fig. 6a; Structural index (SI)) than the control cells (native: *p *= 0.003; fixed: *p *< 10^−5^), indicating that a decrease in migratory activity may correlate with less interaction with the matrix and/or a higher intrinsic contractility expressed through the higher cortical stiffness (Fig. 2) and the F-actin re-arrangement (Fig. 3). On the other hand, although modulating the interaction with the extracellular matrix should be more difficult on a fixed than a native substrate, cells on the fixed substrate displayed a significantly lower SI (*p *= 0.003 and *p *< 10^−4^ for overexpressing and control cells, respectively) and tended to cover a larger area (Fig. 6b, Table 1; *p *= 0.232 and *p *= 0.006 for overexpressing and control cells, respectively native). On both matrices, the area did not differ significantly between NHE1 overexpressing and control cells. Thus, matrix fixation seems to affect cell spreading to a lesser extent than the release of adhesive forces. It is also conceivable that there is a permanent, slightly invasive movement underside, i.e. at the ventral surface of the cells which (i) for technical reasons cannot be observed in 2D experiments such as migration assays on a native substrate and (ii) may not be successful on a fixed substrate. The latter could force the cells to spread and flatten out and thus prevent them from moving deeper into the matrix.

### NHE1 overexpression fuels invasion of native collagen type I

**Fig. 7:**
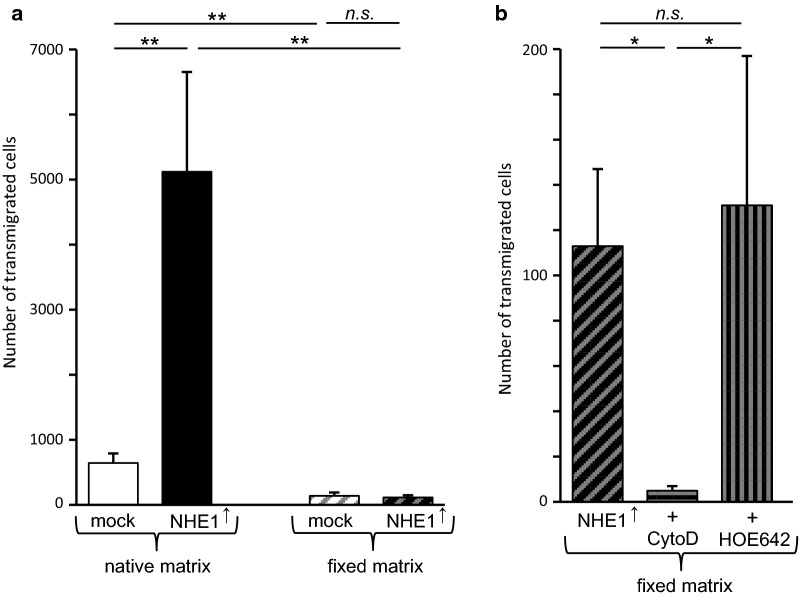
In Boyden chamber assays, MV3 cell invasion is affected by NHE1 expression, matrix fixation and actin cytoskeleton. **a** When seeded on a native matrix, the number of transmigrated cells is considerably higher in NHE1 overexpressing (*N *= 3 with n = 6 filter inserts/wells per experiment) than in control cells (*N *= 3, n = 6). The number of cells crossing a fixed matrix is strongly reduced and does not differ significantly between NHE1 overexpressing (n = 20, *N *= 5) and control cells (n = 14, *N *= 4). **b** On a fixed substrate, NHE1 inhibition by cariporide (HOE642) has no effect on the transmigration of NHE1 overexpressing MV3 cells (n = 15, *N *= 3). Inhibition of actin dynamics with cytochalasin D blocks invasion of NHE1 overexpressing cells almost completely (n = 11, *N *= 3)

**Fig. 8:**
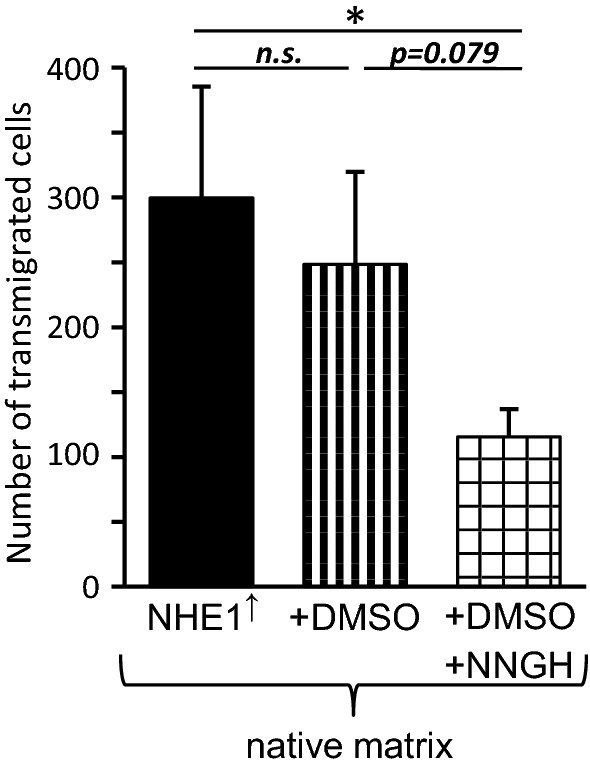
Invasion of NHE1 overexpressing MV3 cells depends on MMP activity. In presence of the MMP inhibitor NNGH in DMSO, the number of cells crossing a native substrate is clearly reduced (n = 6 filter inserts from *N *= 3 independent experiments each)

When observed on a native collagen type I substrate in transwell invasion assays, the NHE1 overexpressing cells were considerably more invasive than the control cells (Fig. 7a; 5120 ± 1533 vs. 644 ± 147 out of originally 200,000 seeded cells transmigrated, *p *= 0.005). Seeded on a fixed substrate, the invasiveness of both control (138 ± 52 transmigrated cells) and NHE1 overexpressing cells (113 ± 34) was strongly reduced while the number of transmigrated cells did not differ (*p *= 0.66). Interestingly, invasion of the fixed substrate did not need NHE1 activity but clearly required actin dynamics, because the NHE1 specific inhibitor cariporide (HOE642) had no effect (131 ± 66 transmigrated cells), whereas cytochalasin D, a potent inhibitor of actin polymerization, blocked invasion nearly completely (5 ± 2 invasive cells (Fig. 7b). In a separate set of experiments, we found that invasion of NHE1 overexpressing cells into the native matrix was reduced by more than 50% in the presence of 10 µmol l^−1^ of the MMP-inhibitor NNGH (Fig. 8). While the number of transmigrated cells was 300 ± 86 in untreated and 248 ± 71 in cells exposed to DMSO, the solvent for NNGH, it reached only 115 ± 22 in cells treated with the MMP-inhibitor. Taken together, the results of the invasion assays suggest that NHE1 overexpression facilitates MMP-dependent invasion of a native substrate while invasion of a fixed substrate is not affected by NHE1 inhibition but requires actin dynamics.

### NHE1 expression stimulates secretion of MMP3

**Fig. 9:**
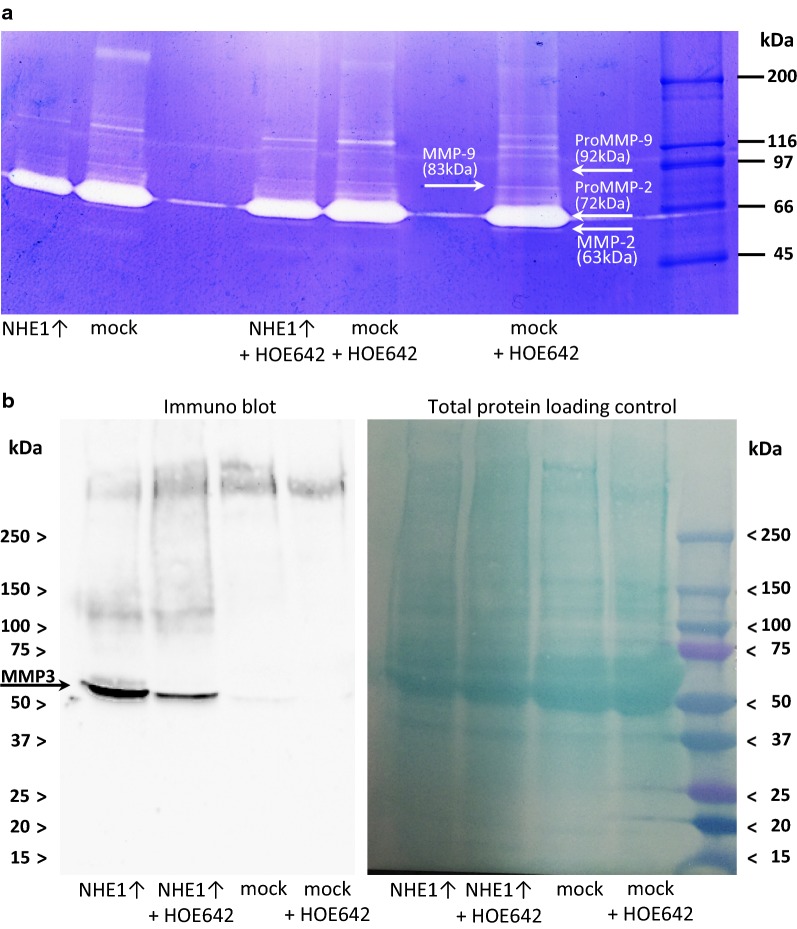
NHE1 overexpression promotes MMP3 secretion in MV3 cells. Supernatants of cell cultures were analyzed. **a** Gelatin degradation assays indicate slight MMP9 and strong pro-MMP9 activity under all conditions examined (NHE1 overexpression, mock control, NHE1 inhibition with cariporide (HOE642)), (*N *= 3). **b** Immunoblot analysis shows a strong secretion of MMP3 (~ 54 kDa) in NHE1 overexpressing cells which can be reduced by cariporide (HOE642) (*N *= 4)

Since MMPs have been shown to play a major role in NHE1-dependent invasion [27, 29], and because in the present study, the MMP-inhibitor NNGH inhibits invasion of MV3 cells, we assessed the activity of MMPs released by the cells into the culture medium by performing zymographic assays. Gelatin degradation assays did not show an NHE1-associated increase in MMP2-, proMMP9- or MMP9-activity (Fig. 9a). Also, casein degradation assays did not show any differences in MMP10-activity (data not shown).

Because MMP3 activates the collagenases MMP1, MMP8 and MMP13 [30] that are capable of degrading collagen types I, II, III, V and IX as well as native fibrillary collagen [31] which is crucial for melanoma metastasis, we tested for MMP3 secretion by performing western blot analyses (Fig. 9b). NHE1 overexpressing cells, as opposed to the mock control, secreted a substantial amount of MMP3. MMP3 secretion was reduced in cariporide (HOE642)-treated cells, indicating that both NHE1 expression and its activity promote MMP3 secretion.

## Discussion

The main purpose of the present study was to check a possible relationship between NHE1 expression, the cortical cell stiffness of human melanoma (MV3) cells and their ability to invade a defined collagen I substrate. We found that the overexpression of NHE1 leads to an increase in cortical stiffness without affecting the bulk stiffness (Fig. 2). This increase in cortical stiffness is accompanied, if not even caused, by a rearrangement of cortical F-actin (Fig. 3). At the same time, although showing an increase in cortical stiffness, the NHE1 overexpressing cells are significantly more invasive (Fig. 7). This increase in invasiveness is probably mediated by an elevated MMP3 secretion and activity (Figs. 8, 9). Since NHE1, especially its activity, stimulates not only the expression of several MMPs at both mRNA and protein level but also their pH-dependent activity [32–34], we minimized the effects of NHE1-mediated MMP activity by utilizing aldehyde-fixed matrices. The cells are hardly able to invade such a fixed collagen substrate. In those cells that do transmigrate across the fixed substrate, the NHE1 inhibitor cariporide (HOE642) has no effect suggesting that NHE1 activity-dependent processes are not crucial under these conditions (Fig. 7). Sound actin dynamics, however, are necessary, as cytochalasin D, an inhibitor of actin polymerization, impedes transmigration across a fixed substrate almost completely.

In the present migration experiments, NHE1 overexpression causes significant cell rounding and slowdown (Figs. 5, 6). The observation that cell rounding accompanied by a decrease in 2D motility correlates with an increased invasiveness is consistent with the finding that murine melanoma (B16V) cells spread and migrate on a basement membrane-like matrix, whereas they hardly migrate on, and instead invade, a dermis-like matrix [35].

NHE1 acts as a structural anchor for actin filaments by directly binding actin binding proteins of the ERM family [9]. Usually, an N-terminal domain of an activated, i.e. phosphorylated, ERM protein binds to a positively charged residue in the cytoplasmic tail of a transmembrane protein, such as NHE1, while its C-terminal domain binds actin filament(s). Thus, ERM proteins cross-link the plasma membrane to the underlying cortical actin [36]. One member of the ERM family is moesin [37]. The activation of moesin upon entry into mitosis is required for cell rounding accompanied by an increase in cortical rigidity [38]. This finding is in line with the present observation that an overexpression of NHE1, one of the binding partners of the ERM family, causes an increase in cortical stiffness associated with a rearrangement of the cortical F-actin.

In addition to its function as a mere structural element, NHE1 may affect cortical stiffness also by its activity. Comparing the cortical stiffness of completely untreated NHE1 overexpressing MV3 cells with that of cariporide-treated NHE1 overexpressing cells does not reveal a significant difference. However, the cortical stiffness of cells treated with the solvent DMSO alone is slightly higher than that of cells treated with cariporide solved in DMSO. Without any doubt, DMSO has a strong impact on the plasma membrane. It induces membrane thinning, increases the fluidity of the membrane’s hydrophobic core and, at higher concentrations, creates transient water pores in the membrane [39]. DMSO as a component of freezing media causes a significant increase in the stiffness of mouse embryonic fibroblasts [40]. Moreover, DMSO increases the membrane permeability for K^+^ in a dose-dependent manner in monocytes (THP-1 cells [41]). The diameter of a hydrated K^+^ ion is assumed to be ~ 0.133 nm while that of a hydrated Na^+^ ion comes to ~ 0.5 nm (0.095 nm non-hydrated ionic radius + ~ 0.4 nm hydration shell [42]). Nonetheless, it could be possible that DMSO increases the membrane permeability for Na^+^ as well. The DMSO-mediated increase in the membrane permeability for water and/or Na^+^ would lead to an osmotic swelling resulting in a higher stiffness. Provided that unfolding membrane reservoirs are not available, osmotic swelling would cause the cell membrane to stiffen [43] while, at the same time, the cortical actin cytoskeleton would behave like an expanding sponge and enhance the stiffening [44]. In the present study, cariporide (HOE642) in presence of its solvent DMSO reduces the cortical stiffness. At this point, we are not able to determine to what extent (i) a decrease in the number of Na^+^ ions imported by NHE1 and thus osmotic shrinkage [45, 46], (ii) a decrease in cytosolic pH [47] or (iii) other factors such as pH-dependent signaling [48] contribute to the cariporide-induced, slight decrease in cortical stiffness.

The restructuring of the cortical actin meshwork accompanied by an increase in cortical stiffness (Figs. 2, 3) most likely modulates both the architecture including the lipid packing [49] and the composition of the plasma membrane [50] of NHE1 overexpressing cells. This could then modulate enzymatic activities [51] and catalyze the conversion of sphingolipids such as ceramide, sphingomyelins or glycosphingolipids [52, 53]. For instance, the acid sphingomyelinase catalyzes the cleavage of sphingomyelin to produce ceramide and phosphorylcholine, and the sphingomyelin deacylase catalyzes the hydrolysis of *N*-acyl-sphingosylphosporylcholine leading to the generation of a fatty acid and sphingosylphosphorylcholine (SPC). SPC induces the expression and secretion of MMP3 [54]. In the present Western blot analysis, NHE1 overexpression in MV3 cells is accompanied by an increase in MMP3 secretion. Normally, at physiological pH values of ~ 7.4, NHE1 activity and thus the number of H^+^ delivered to the cell surface are rather low. However, not only the acid sphingomyelinase [55] but also the sphingomyelin deacylase [56] show their maximum activities at rather low pH values of pH ~ 5.0. Since we found an increased MMP3 secretion in the NHE1 overexpressing cells and because in a wide variety of cancers including melanoma cells NHE1 activity is considerably elevated [1, 3, 57, 58], it is conceivable that the number of H^+^ ions released at the cell surface may be high enough to sufficiently stimulate the sphingomyelin-converting enzymes (and through SPC indirectly MMP3) even though neither the proper nor a large-area pH optimum is reached. Immunoblotting the media revealed that NHE1 overexpressing MV3 cells do secrete more MMP3 depending on NHE1 activity (Fig. 9b). Furthermore, MMP3 as activator of collagenases [30] most likely plays a crucial role because MMP inhibition by NNGH leads to a distinct decrease in transmigration across native matrices (Fig. 8).

Both expression and activity of NHE1 affect the cell cycle and correlate with proliferation [59, 60]. Therefore, the increase in the number of MV3 cells that invade native matrices could be partially due to an increased proliferation associated with NHE1 overexpression (Fig. 7a). On the other hand, this assumption should hold true also for NHE1 overexpressing cells invading a fixed matrix. But there is no difference in transmigration between NHE1 overexpressing and control cells.

Fixation of extracellular matrices with glutaraldehyde modifies their micro-elastic properties and leads to a substantial increase in matrix stiffness [61]. The matrix stiffness modulates cell behavior [62], induces malignant phenotypes [63] and can trigger epithelial–mesenchymal transition (EMT [64]). In fact, the MV3 cells are more spread and less spherical on a glutaraldehyde-fixed compared to a native collagen type I substrate. While unchanged in control cells, cell motility of NHE1 overexpressing MV3 cells is significantly decreased on a fixed substrate (Fig. 5). Albeit this is probably caused by the absence of H^+^-dependent events such as modulation of pH-sensitive cell/matrix interactions [6] and MMP activity [34], a certain impact of the matrix rigidity on cell motility cannot at all be excluded and could also affect transmigration across the fixed substrate. In a three-dimensional setting such as the Boyden chamber/transmigration assay it is hardly possible to precisely dissect to what extent the different physiological and biophysical parameters affect invasiveness.

## Conclusion

Given the entire data set, we conclude that in MV3 cells, NHE1 overexpression causes a rearrangement of F-actin at the cell cortex associated with an increase in cortical stiffness. This rearrangement in F-actin is likely to rely on the pH-sensitive and thus NHE1-dependent interaction between cortactin and cofilin [65]. Despite their higher cortical stiffness NHE1 overexpressing cells are considerably more invasive on a collagen type I substrate, most likely due to increases in MMP3 secretion and activity. This is in line with the proposed concerted roles of cortactin and F-actin in regulating the secretion of proteases [66]. In addition, the link between NHE1 overexpression and MMP3 secretion may also include the architecture and composition of the plasma membrane and should be analyzed in future studies. In the long term and with simultaneous consideration of the (cellular) lipid metabolism, the relationship between the expressions of NHE1 and MMP3 may gain in importance for the development of anticancer drugs.

## Data Availability

The datasets used and/or analysed during the current study are available from the corresponding author on reasonable request.

## Abbreviations

B16V: murine melanoma cell line
DMSO: dimethyl sulfoxide
ECM: extracellular matrix
ERM: ezrin, radixin, moesin protein family
EMT: epithelial-to-mesenchymal transition
HOE642: cariporide (NHE1 inhibitor)
MMP: matrix metalloproteinase
MV3: human melanoma cell line
NHE1: Na^+^/H^+^ exchanger 1
NNGH: *N*-isobutyl-*N*-(4-methoxyphenylsulfonyl)glycyl hydroxamic acid
PBS: phosphate buffered saline
PVDF: polyvinylidene difluoride
SDS: sodium dodecyl sulfate
SPC: sphingosylphosphorylcholine
TBS-T: Tris-buffered saline with Tween20
Tris: Tris(hydroxymethyl)-aminomethane
